# Repression of Carotenoid Accumulation by Nitrogen and NH_4_^+^ Supply in Carrot Callus Cells In Vitro

**DOI:** 10.3390/plants10091813

**Published:** 2021-08-31

**Authors:** Tomasz Oleszkiewicz, Michał Kruczek, Rafal Baranski

**Affiliations:** Department of Plant Biology and Biotechnology, Faculty of Biotechnology and Horticulture, University of Agriculture in Krakow, 31-425 Krakow, Poland; kruczek.michael@gmail.com (M.K.); rafal.baranski@urk.edu.pl (R.B.)

**Keywords:** *Daucus carota*, carotene, nitrate, ammonium, somatic embryogenesis

## Abstract

The effect of mineral nutrition on the accumulation of the main health beneficial compounds in carrots, the carotenoid pigments, remains ambiguous; here, a model-based approach was applied to reveal which compounds are responsible for the variation in carotenoid content in carrot cells in vitro. For this purpose, carotenoid-rich callus was cultured on either BI (modified Gamborg B5) or R (modified Murashige and Skoog MS) mineral media or on modified media obtained by exchanging compounds between BI and R. Callus growing on the BI medium had abundant carotene crystals in the cells and a dark orange color in contrast to pale orange callus with sparse crystals on the R medium. The carotenoid content, determined by HPLC and spectrophotometrically after two months of culture, was 5.3 higher on the BI medium. The replacement of media components revealed that only the N concentration and the NO_3_:NH_4_ ratio affected carotenoid accumulation. Either the increase of N amount above 27 mM or decrease of NO_3_:NH_4_ ratio below 12 resulted in the repression of carotenoid accumulation. An adverse effect of the increased NH_4_^+^ level on callus growth was additionally found. Somatic embryos were formed regardless of the level of N supplied. Changes to other media components, i.e., macroelements other than N, microelements, vitamins, growth regulators, and sucrose had no effect on callus growth and carotenoid accumulation. The results obtained from this model system expand the range of factors, such as N availability, composition of N salts, and ratio of nitrate to ammonium N form, that may affect the regulation of carotenoid metabolism.

## 1. Introduction

The carrot is a well-known vegetable grown around the world for its nutritious storage root. The roots of the most common carrot varieties accumulate carotenoids, mainly β-carotene and α-carotene, which give them their orange color. Both carotenes have provitamin A activity and, together with other carotenoids, play beneficial roles in human health [1]. The high carotene content makes carrots one of the most important sources of carotenoids in the human diet [2], and knowledge on carotenoid biosynthesis, accumulation, and regulation of these processes is essential for the development of high-quality carrot varieties.

Carotenoids exist widely in nature. They are 40-carbon molecules built from eight base isoprenoid units. They are classified to two main groups: carotenes, being hydrocarbons such as β-carotene and α-carotene, and xanthophylls, which are oxidized carotenes [3]. The processes of carotenoid biosynthesis in plants, including carrot, have been well described [4]. In recent years, research has focused on understanding the regulation of biosynthetic pathway and carotenoid sequestration. Currently, it is known that developmental and environmental factors, such as light, influence carotenoid accumulation in carrot cells [5,6]. Field conditions and genotype have a pronounced effect on carotenoid accumulation in carrot storage roots in contrast to plant fertilization [7]. However, fertilization with calcium ammonium nitrate increased carotenoid content [8]. The effect of other N salts was equivocal unless an additional foliar nutrition with a complex fertilizer was applied [9]. Variation in carotenoid content among carrot cultivars was also reported depending on the applied urea dose [10]. Thus, the conclusions regarding the effect of nutrition on carotenoid metabolism in carrot remain ambiguous. Recently, it was demonstrated that ammonium ions negatively affect carotenoid accumulation in *Calendula officinalis* callus cultured in vitro [11]. Another genetic research based on callus response to changes in the composition of mineral medium indicated that N supply affected pigment accumulation [12].

For more than 60 years, the carrot has been considered a model species in research on totipotency, somatic embryogenesis, and horizontal gene transfer (for review see [13] and [14]), while broad genetic research, including the recent genome sequencing project [15], led to the development of high-quality varieties [16]. Carrot is amenable to cell and tissue culture on mineral media in vitro. Callus can be induced in in vitro culture from various explants by the supplementation of mineral medium with growth regulators and then it can be easily propagated [17], hence, it has become a convenient material for research on stress factors, genetic transformation, and genome editing, including genes of the carotenoid pathway [18,19]. Carrot callus usually accumulates low amounts of carotenoids, as do the storage root meristematic cells used to induce callus [20,21]. However, the development of carotenoid-rich callus was also reported [22,23,24], and recently, it has been successfully used for structural studies of carotene crystals [25,26], regulation of carotenoid biosynthesis, sequestration, and interaction of carotenoid and cell wall composition when it was subjected to targeted mutagenesis using novel tools of genome editing, i.e., clustered regularly interspaced short palindromic repeats (CRISPR) and CRISPR associated (Cas9) proteins [19,24].

Inorganic components in the medium are necessary for the growth of plant tissues and organs in vitro. Their contents and composition influence tissue and plantlet development, hence, the proper balance of medium components is required [27]. For the induction of carrot callus development, and for its further propagation, a medium based on Gamborg B5 [28] mineral salts and vitamin composition is more effective and more often used than the Murashige and Skoog (MS; [29]) medium [30]. The MS medium is recommended for somatic embryogenesis and carrot plant regeneration [31,32]. Our preliminary experiments showed that attempts of plant regeneration using a modified MS medium, the R medium, led to a visually paler color of callus due to a reduced carotenoid accumulation in comparison to callus grown on the B5-based BI medium. Both media, BI and R, differ in the composition of salts, vitamins, plant growth regulators, and in the sucrose concentration. Thus, in this study we sought for an answer to which component of the R medium is responsible for the repression of carotenoid accumulation. For this purpose, we used a model carotenoid-rich callus [23] and exposed it to media with modified compositions. A substantial reduction of carotenoid content was observed on the B5 medium with altered composition of nitrogen salts, thus, we show here that the amount of N and NO_3_:NH_4_ ratio are key factors affecting carotenoid accumulation in carrot cells.

## 2. Results

### 2.1. Callus Growth and Morphology

**Figure 1:**
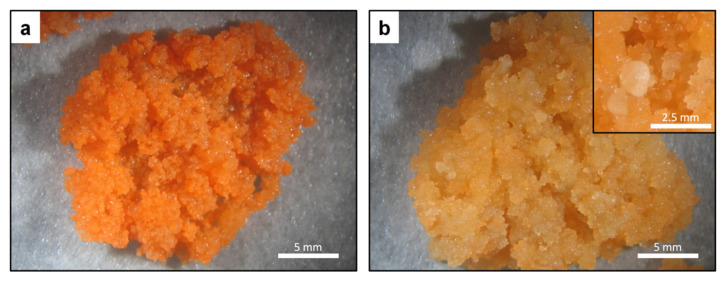
Carrot callus growing on mineral media. (**a**) BI medium; (**b**) R medium; (**b-inset**) embryo-like structure.

**Figure 2:**
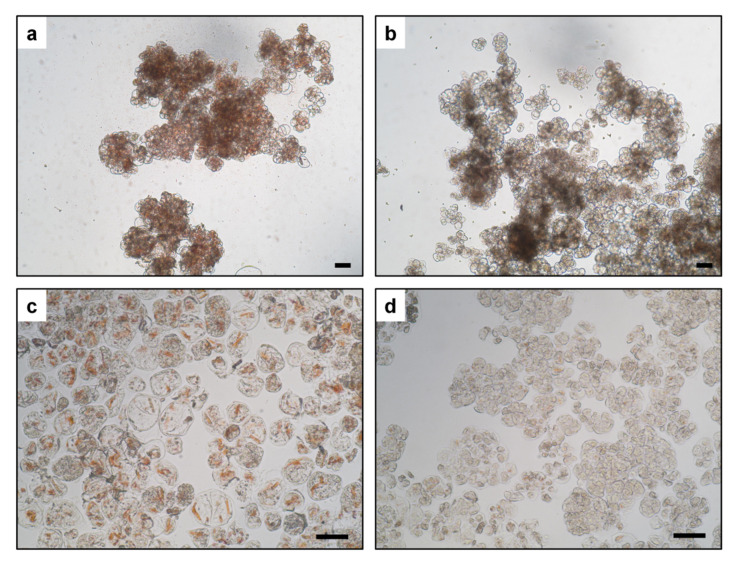
Densely packed carrot callus cells (not macerated tissue) growing on either the BI (**a**) or R medium (**b**). Easily noticeable carotene crystals in cells growing on the BI medium (**c**) and sparse crystals in cells growing on the R medium (**d**). Bar = 100 µm.

Carrot callus cultured on both BI (modified Gamborg B5) and R (modified MS) media (Supplementary Table S1) grew with a similar rate. Callus cultured on the BI medium retained its characteristic morphology throughout all experiments. It had dense and lumpy structure, with small parts being more friable. It retained orange color, although rarely, friable callus was paler (Figure 1a). Callus growing on the R medium differed from callus on the BI medium in color, which became paler and eventually light orange over the course of time (Figure 1b). Microscopic observations revealed that regardless of the medium, callus cells were densely packed in aggregates (Figure 2a,b). Carotene crystals, clearly distinguishable due to their intense orange color, were sequestered in the cells of dark orange callus on the BI medium (Figure 2c). Cells of callus maintained on the R medium contained only small crystals, and they were not abundant (Figure 2d). Additionally, proembryogenic tissue was identified in callus on the R medium. Embryoid structures were visible (Figure 1b inset); however, their development was arrested at early stages. They did not convert into plants, turned brown, and often died or dedifferentiated to new callus cell layers.

### 2.2. Carotenoid Content in Callus

**Figure 3:**
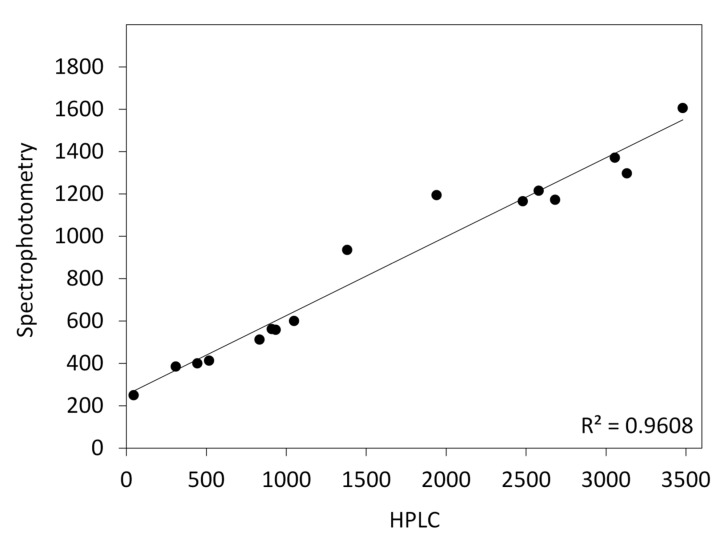
Fitted linear regression model for carotenoid content (µg/g DW) in carrot callus, determined based on HPLC (sum of α- and β-carotene) and spectrophotometric measurements (total carotenoids).

Two main carotenes were identified in callus using HPLC (Table 1 and Supplementary Figure S1). The sum of α- and β-carotene contents in callus growing on the BI medium was very high (2264 µg/g DW) and exceeded that in callus on the R medium (425 µg/g DW) by 5.3 times, which corresponded to differences in color observed between calli on both media. The β/α carotene ratio (2.6) was also higher for the BI medium than for the R medium (1.5) (Table 1). The sum of α- and β-carotenes determined by HPLC highly correlated (*r* = 0.98, *p* < 0.001) with the total carotenoid content, determined spectrophotometrically, although it was usually higher. A linear relationship was described by a well fitted regression line (*p* < 0.001) with the coefficient of determination R^2^ = 0.96 (Figure 3). Hence, quantitative determination of carotenoid content in further experiments was done using spectrophotometry.

### 2.3. Effect of Medium Composition

To identify compounds that affected the carotenoid content in callus, 12 media varying in the composition of main compound groups were compared. For this purpose, the composition and amounts of macroelements, microelements, vitamins, growth regulators, or sucrose in the BI medium were replaced by the corresponding compound groups, and in the same amounts, as present in the R medium (Table 2). Analogous modifications were applied to the R medium by replacing compound groups to be the same as in the BI medium. Callus growing on the modified media showed changes in color and carotene contents (*p* < 0.001). The replacement of macroelements in the BI medium caused callus discoloration and the decrease of carotenoid content by 54.6% to a level similar to the R medium (Table 2). The effect of B5 macroelements added to the R medium was also significant. Callus growing on the R/B5-macro medium had an intense orange color and over 3-fold increased carotenoid content in comparison to the unmodified R medium, reaching the carotenoid level present in callus on the BI medium (Table 2). Thus, the Gamborg B5 composition of macroelements stimulated carotenoid accumulation in contrast to the MS formulation of macroelements. Any other changes done to either the BI or R media composition (microelements, vitamins, growth regulators, sucrose) did not significantly affect carotenoid content (Table 2). These results indicated that macroelement composition in the medium was critical for carotenoid accumulation in callus. The only other effect of modified media was the formation of embryoid structures on the BI medium free of 2,4-D and kinetin that resembled structures observed in callus on the R medium.

### 2.4. Effect of Macroelements

Various salts of macroelements were present in the BI and R media, thus, any element or their combination could affect carotene content. The contents of individual macroelements in the BI medium were modified to get the same molar concentrations as in the R medium. Modifications to either P, K, Ca, or Mg contents did not result in changes either of callus morphology, color, or carotenoid content. A noticeable callus discoloration was observed only on the medium with a modified composition of N salts. Callus exposed to the BI/MS-N medium developed more white or pale orange cell aggregates. It accumulated almost 40% less carotenoids than callus on the BI medium and had similar amounts of carotenoids as callus on the R medium (Table 2). The N content depends on the amounts of NH_4_NO_3_ and KNO_3_ salts, and KNO_3_ supplementation results in the increased concentration of both N and K. To verify the effect of K on carotenoid content, the R and BI/MS-N media were supplemented with K_2_SO_4_ (R+K and BI/MS-N+K, respectively). No effect of additional K amounts on carotenoid level was found.

### 2.5. Effect of N Concentration and NO_3_:NH_4_ Ratio

**Figure 4:**
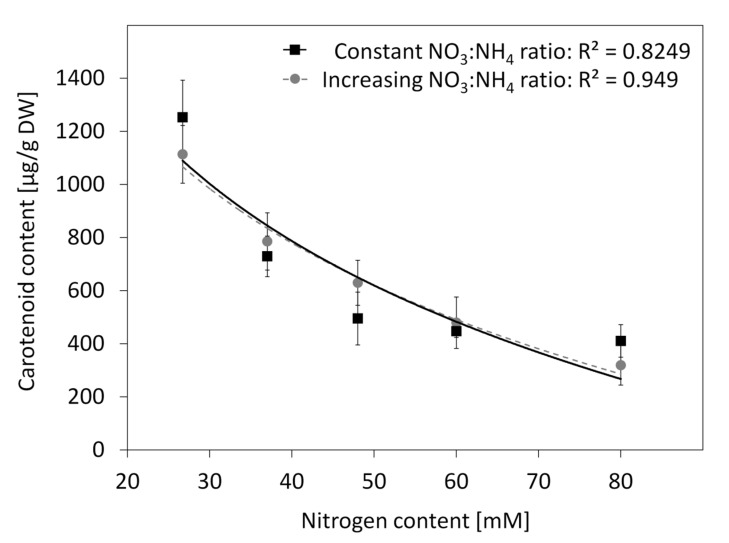
Carotenoid content in callus grown on the BI media with modified N content and with the constant (12.2) NO_3_:NH_4_ ratio (squares, solid line) or with increasing NO_3_:NH_4_ ratio from 12.2 to 38.5 (dots, dashed line). Lines represent logarithmic functions: y = 3551.5 − 749.5ln(x) for the constant NO_3_:NH_4_ ratio and y = 3852 − 820.7ln(x) for the increasing NO_3_:NH_4_ ratio; whiskers—std. error.

The N content in the R medium (60.02 mM) was more than doubled in comparison to the BI medium (26.76 mM) (Table 3). To verify the effect of N concentration on carotenoid accumulation, media differing in composition of N salts were compared (Supplementary Table S2). The increase of N amount in the range from 27 mM to 80 mM did not affect callus growth but significantly reduced carotene content from 1252 µg/g DW to 411 µg/g DW. Such changes were highly significant independent from whether the NO_3_:NH_4_ ratio in all comparing media was the same, i.e., 12.19 (the same as in the BI medium) or it was increasing in the range from 12.19 up to 38.5 (both *p* < 0.001). In both sets of media, the observed reduction of carotene content followed similar trends described by logarithmic functions with R^2^ of 0.8249 and 0.9490, respectively (Figure 4).

**Figure 5:**
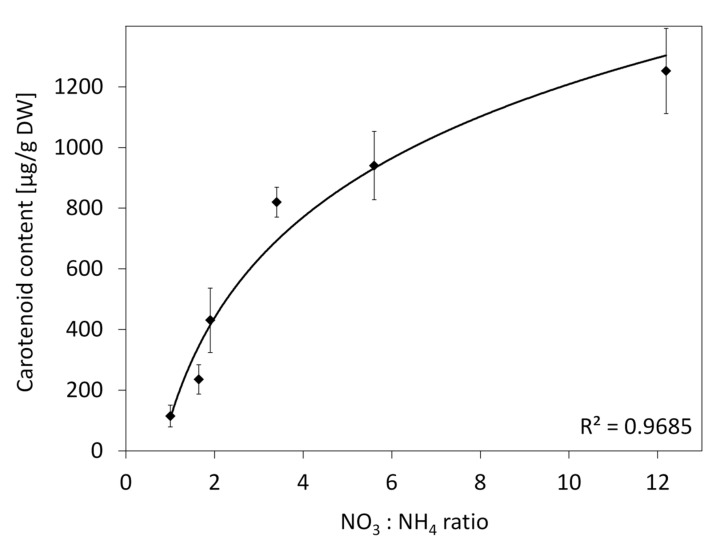
Carotenoid content in callus grown on BI media with a modified NO_3_:NH_4_ ratio and with the constant N content (26.8 mM). The line represents a logarithmic function y = 107.65 + 478.27ln(x); whiskers—std. error.

The R medium contained NH_4_NO_3_ not present in the BI medium, which had NH_4_^+^ ions supplied in a low amount of (NH_4_)_2_SO_4_ not present in the R medium (Table 3). In consequence, the R medium had more N, mainly due to the use of an amount 10.2 times higher of the ammonium form while the nitrate form was only 1.6 times higher. The effect of N form in the medium on the carotene content was verified by using media containing 26.76 mM N, the same as in the BI medium, adjusted by using both nitrate and ammonium salts in various ratios. While decreasing the NO_3_:NH_4_ ratio down to 1.91 (the same as in the R medium), callus grew similar to the callus exposed to the BI medium with the NO_3_:NH_4_ ratio of 12.19. Further elevation of the NH_4_^+^ amount in the media restricted callus growth, which was eventually inhibited on the medium with the 1:1 NO_3_:NH_4_ ratio. The increasing NH_4_^+^ level also highly reduced carotenoid content in callus (*p* < 0.001). In comparison to the BI medium, the carotenoid content was reduced 3.0-fold at the NO_3_:NH_4_ ratio of 1.91 (the same as in the R medium), and a further increase of NH_4_^+^ to the 1:1 NO_3_:NH_4_ ratio reduced the carotenoid content by 11.5-fold to the level of 115 µg/g DW. Such changes in the carotenoid content followed a trend described by a well fitted logarithmic function with R^2^ = 0.9685 (Figure 5).

## 3. Discussion

Nutrient supply and their uptake by plants determine yield and quality of agricultural products, including carrot, and available data indicate that fertilization, in particular with N, may also affect carotenoid accumulation in carrot storage roots. Previous evaluations of NPK fertilization showed that genotype and environmental conditions affected carotenoid accumulation rather than N supply [7]. These conclusions were supported by results of a multiyear field trial using various N fertilizers, although significant increase of carotenoid content was achieved after foliar nutrition [9]. Fertilization with urea suggested variation in carotenoid content in used cultivars depending on the urea dose, although differences between overall means were insignificant [10], while fertilization with calcium ammonium nitrate increased carotenoid accumulation in two cultivars in a two-year trial [8]. The conclusions of field studies on plant nutrition remain ambiguous as the results are highly affected by complex environmental factors, additionally interacting with variety.

Experiments utilizing cell and tissue culture in vitro allow to apply controlled conditions that, in particular, are essential in plant nutrition research, and which are not possible to obtain in field conditions. Therefore, we have applied a research model to elucidate at cellular level the role of nutrition on the accumulation of the main carrot health beneficial compounds, the carotenoid pigments. The MS-based mineral media had already been used to culture cell suspension or to induce callus for carotenoid research. The cell suspension or callus from a red storage root carrot variety accumulated mainly β-carotene and lycopene; the level of these pigments highly varied and was clone-dependent [33,34]. For *Arabidopsis thaliana*, the carotenoid content in wild type callus cultured on the medium containing MS salts was low, 200–550 µg/g DW [35]. For *Tagetes erecta* [36], individual carotenoids were identified and not quantified, but the assessment of color and HPLC profiles also indicated low amounts of pigments. The Gamborg B5-based mineral media were used to induce development of light-orange [24] and dark-orange, carotenoid-rich, carrot callus accumulating up to the same amounts of carotenoids (2150 µg/g DW) as the storage root from which such callus was derived [23]. Thus, carotenogenesis was ongoing in materials cultured on the MS-based media, but the B5-based media were much more efficient for pigment accumulation. The observed color variation of carrot callus cultured on different mineral media in vitro have indicated that accumulation of carotenoid pigments is stimulated or repressed by media components. The mineral compositions of BI and R media, used in this work, differed significantly as they were essentially based on the Gamborg B5 and MS formulations, respectively. Both media differed mainly in their N salts composition. The amount of N was 2.24 times higher in the MS medium, and N was supplied in NH_4_NO_3_ and KNO_3_ salts, of which the former was present in a higher concentration, thus, the NO_3_:NH_4_ ratio in MS was 1.91 (Table 3). The B5 medium was richer in the nitrate salt by 32% but contained a low amount of ammonium (NH_4_)_2_SO_4_ salt, thus, the NO_3_:NH_4_ ratio in B5 was 12.19. Hence, the B5 medium contained 10.2 times less ammonium N form than MS. In this study, we found that callus grown on the BI medium and on any modified BI medium containing N salts according to the Gamborg B5 formulation accumulated many more carotenoids than when using MS-based N salts.

Both BI and R media differed also in the composition of other compounds. The MS medium contained three times more CaCl_2_, 50% more MgSO_4,_ and had KH_2_PO_4_ instead of NaH_2_PO_4_. Although these differences are less pronounced than differences in N salts composition, they were also taken into account in this study. It was previously reported that a reduction of Ca supply promoted carotenoid accumulation in the roots of carrot plants, but this effect was variety dependent, with the most significant effect on lycopene content in a lycopene accumulating variety [37]. When using a lycopene accumulating carrot cell suspension, it was shown that increasing the initial P content in the medium or resupplying P during the culture increased carotenoid accumulation [38]. It was also shown that the increase of 2,4-D up to 10 ppm promoted carotenoid accumulation in carrot cells [33]. A higher sucrose concentration increased carotenoid content, with 3%−5% sucrose being optimal, while 8% sucrose had adverse effects on carrot cells growth and their size [39]. Additionally, the carotenoid accumulation increased in *Calendula officinalis* callus when sucrose concentration was raised from 4% to 7% [11]. In our work, no significant changes in carotenoid accumulation in carrot callus was found when modifying the compositions of Ca, P, K, and Mg salts. Further BI medium modifications by replacing microelements, vitamins, elimination of growth regulators, and reduction of sucrose from 3% to 2%, as present in the R medium, had no significant impact. No response of carrot callus to all these modifications supports the conclusion that N availability is the prime factor affecting carotene accumulation. This finding is in contrary to the results presented by Hanchinal et al. [40], who modified N, P, and sucrose concentrations and used a response surface methodology to optimize β-carotene production by carrot cells in suspension. A doubled N concentration to 50 mM with increased sucrose content from 2% to 3% increased β-carotene production up to 13.61 µg/g DW. However, it must be underlined that they used cells accumulating very low amounts of carotenoids, two magnitude lower than callus in our work, which may highly bias the conclusions.

Our results showed also that a gradual increase of N from 26.76 mM to 80.04 mM restricts carotenoid accumulation, which eventually decreased 3-fold. Nitrogen was supplied mainly in the form of KNO_3_, thus, the amounts of N and K in the medium were interrelated. Further media adjustments with K salts to keep this element at the same level while increasing N concentration showed that K did not affect carotenoid accumulation, confirming that the N amount in the medium is a critical factor and, moreover, its effect is independent on the NO_3_:NH_4_ ratio in the range from 12.2 to 38.5. Additionally, the amount of N did not alter callus growth. Recent study on grape callus showed that the reduced N amount in the MS medium from 60 mM to 40–50 mM enhanced accumulation of other pigments, anthocyanins, in red-pod okra callus; however, further reduction to 30 mM had an adverse effect [41]. Additionally, N starvation promoted anthocyanin accumulation in grape callus [42]. In contrary, other reports showed that the increase of total N content by doubling KNO_3_ in the MS medium increased anthocyanin content by 135% [12], which was congruent with results showing the highest accumulation of anthocyanins using an elevated N amount (70 mM) in the medium [43].

The N content in a medium depends on the combination of supplied ammonium and nitrate salts. The ammonium N form is preferred by plants as it can be directly used, and its incorporation by a cell requires less energy. However, it can become toxic to plant cells at higher concentrations, and plant sensitivity to ammonia varies greatly depending on species, plant age, and environment pH [44]. Hairy roots of carrot, red beet, and madder in the presence of NH_4_^+^ available in the amounts in the MS medium had a reduced growth [45]. A similar effect of restricted growth was observed for anthocyanin accumulating carrot callus cultured on MS [12]. No growth changes were reported only when carrot cell suspension was exposed to a doubled amount of ammonium N form than present in MS [46]. The comparison of a wide range of NO_3_:NH_4_ ratios in our work demonstrates that when keeping the optimum N level (26.76 mM) for carotenoid accumulation, as in the B5 medium, the callus growth is restricted with increasing amounts of NH_4_^+^, as is the carotenoid content. This adverse effect of the ammonium form led to an over 10-fold reduction of carotenoids and such response intensified logarithmically with the NH_4_^+^ concentration. A similar adverse effect of high NH_4_^+^ concentration was found in *Calendula officinalis* callus. The 50% decrease of NH_4_^+^ amount, in comparison to MS, induced carotenoid biosynthesis, and the complete removal of NH_4_^+^ from the medium further promoted carotenoid accumulation [11]. An analogous effect of increasing NH_4_^+^ concentration was reported in relation to anthocyanin accumulation. Lower anthocyanin contents were recorded in carrot cells in suspension exposed to media with a low NO_3_:NH_4_ ratio, and the increase of the ratio to 4:1 led to the highest pigment content [43]. In callus induced from rose leaves, the reduction of NH_4_^+^ and increase of NO_3_^−^ concentrations in the MS-based medium enhanced anthocyanin accumulation [47]. Conversely, doubling the concentration of NH_4_^+^ in the MS medium restricted the anthocyanin content by one third in carrot callus [12].

Nitrogen is required by plants for their growth, but the composition and concentrations of N salts in a culture medium affect also morphogenesis and embryogenesis [27]. It was previously shown that a reduced form of N, present in high amounts in MS, is required for the development of somatic embryos from carrot hypocotyl explants. Three N salts with reduced N were compared and two of them, NH_4_NO_3_ and NH_4_Cl, favored somatic embryogenesis, while (NH_4_)_2_SO_4_ did not [48]. However, contrary to these results, nearly 20 times more plants developed on the B5 medium, which contained much less of the reduced N form and was supplied with (NH_4_)_2_SO_4_ salt only, than on the MS-based medium [49]. The comparison of several media free of growth regulators in our work has shown that the formation of proembryogenic tissue and globular embryos is ongoing regardless of the medium mineral composition. Somatic embryos were observed on both, carotenoid-rich callus and low carotenoid accumulating callus, thus, there was no clear relationship between somatic embryogenesis and N salt composition, hence, carotenoid accumulation, although quantitative comparison was not the subject of this work.

## 4. Materials and Methods

### 4.1. Plant Material, Media Preparation, and Experiment Design

Dark-orange callus derived from the root of DH1 (doubled haploid) carrot (*Daucus carota* L.) line accumulating high amounts of carotenoids and described previously [23] was used. Callus was maintained on filter paper disks laid down on the surface of solidified mineral medium in 9 cm Petri dishes and cultured at 26 °C in the dark. In each experiment, callus was grown for eight weeks with one transfer to a fresh medium after four weeks. Two main media were used: (1) the BI medium consisting of Gamborg B5 macro- and microelements with vitamins, 30 g/L sucrose, 1 mg/L 2,4-D, and 0.0215 mg/L kinetin, and (2) the R medium consisting of MS macro- and microelements with vitamins (with an increased glycine content to 3 mg/L), 20 g/L sucrose, and free of growth regulators (Supplementary Table S1). Both media had pH adjusted to 5.8 and were solidified with 2.7 g/L phytagel. Macro- and microelements, including vitamin mixtures, separate macro- and microelement mixtures, vitamins, and plant growth regulators were purchased from Duchefa Biochemie (Haarlem, The Netherlands). Media modifications were done by exchanging group of components between BI and R media (12 media variants; Table 2), or by exchanging individual compounds (eight variants; Table 2), or by changing nitrogen salts composition and their concentration (14 variants) (Supplementary Table S2).

### 4.2. Microscopic Observations

To observe callus structure, small callus pieces were placed on a microscopic slide in a water drop under the cover slide. Carotenoid crystals were observed in single cells after tissue maceration in 1N HCl at 50 °C for 5 min. Observations were done in a bright-field using the Zeiss Axiovert S100 microscope with ×10 objective. Images were collected by using the attached digital camera.

### 4.3. Determination of Carotenoid Content

Eight-week-old callus was lyophilized and ground into a fine powder in a beading mill for 5 min. Carotenoids were extracted from 5–10 mg samples with 500 µL of acetone in 1.5 mL tubes. Samples were vortexed for 30 s and centrifuged at 18,000 g for 5 min. Acquired extracts were transferred to fresh tubes. The procedure was repeated to ensure complete extraction, and then, obtained extracts were combined. The absorbance of extracts was measured in a 1 cm QS quartz cuvette (Hellma Analytics, Müllheim, Germany) at 450 nm using the NanoDrop 2000c (ThermoScientific, Waltham, MA, USA) spectrophotometer. Extractions and measurements were done for each sample in triplicate and the readouts were averaged before statistical analysis. The total carotenoid content was calculated based on the β-carotene extinction coefficient ($A_{1cm}^{1\%}$ = 2500) using the formula:

$$\frac{\frac{absorbance\;\left(450\;nm\right)}{2500}\times extract\;volume\;\left(ml\right)\times 10000}{sample\;mass\;\left(g\right)}$$

The results are presented in µg of carotenes per gram of callus dry weight.

High performance liquid chromatography (HPLC) measurements were done using the same callus samples as for spectrophotometry. HPLC was performed as previously described [19]. Briefly, the extraction was performed using ethanol:*n*-hexane (1:1, *v*:*v*) and HPLC was performed using Shimadzu LC–20AD chromatograph equipped with a C18 RP (5 µm) column and the Shimadzu SPDM–20A–DAD photodiode-array detector. The identification of β-carotene was based on the retention time of the standard and confirmed by analysis of absorption spectra. The identification of α-carotene was based on the analysis of the absorption spectra. Quantification of β-carotene was done using a standard curve, while α-carotene was quantified in relation to β-carotene.

### 4.4. Statistical Analysis

Each experiment was set up in four replicates, each consisting of five calli, and having a completely randomized design. A one-way ANOVA was performed to test effects of media composition on carotene contents in callus using the Statistica v.13.1 software (TIBCO; Palo Alto, CA, USA). Differences between means were verified at the significance level *p* = 0.05 using the Dunnett test. Means are presented with their standard errors.

## 5. Conclusions

In this study, we sought for the answer to which component of the culture medium affects carotenoid accumulation in carrot callus. A compound by compound replacement in the MS and Gamborg B5 media have revealed that the only critical element is nitrogen, and either the increase of the total N concentration or the decrease of NO_3_:NH_4_ ratio restricts carotenoid accumulation. Thus, the highest carotenoid content was achieved in the medium with 26.76 mM N and 12.19:1 NO_3_:NH_4_ ratio, regardless of whether the other media components were supplied according to the MS or Gamborg B5 formulation. These model-based obtained results pave the way for further elucidation of biological processes related to regulation of carotenoid metabolism. The observed effects might be limited to a simplified in vitro model; hence, further confirmation in planta may be required. However, they can be useful for research or application purposes using cell or tissue culture where stimulation of valuable secondary metabolites, such as carotenoids, is required.

## Figures and Tables

**Table 1:** Carotenoid content [µg/g DW] in callus growing on the BI and R media.

Medium	α-carotene ^1^	β-carotene ^1^	β:α Ratio	Total Carotenoids ^2^
BI (modified Gamborg B5 medium)	627 ± 73 a	1637 ± 305 a	2.6	1169 ± 89 a
R (modified MS medium)	172 ± 55 b	253 ± 113 b	1.5	404 ± 62 b
BI/MS-macro (BI with macroelements as in MS (R))	304 ± 35 b	529 ± 101 b	1.7	531 ± 44 b
R/B5-macro (R with macroelements as in B5 (BI))	725 ± 55 a	2191 ± 182 a	3.0	1322 ± 99 a
BI:R ratio	3.6	6.5	nd ^3^	2.9

^1^ determined by HPLC, ^2^ determined spectrophotometrically; means ± std. error (*n* = 4); means followed by different letters within column are significantly different at *p* = 0.05; ^3^ not determined.

**Table 2:** Modified media used for callus culture and the carotenoid content in callus after eight-week culture (mean ± std. error).

Experiment	Medium	Medium Modification	Carotenoid Content (µg/g DW)	%BI ^1^	*P* (BI) ^2^	*P* (R) ^2^
Modification of compound groups	BI ^3^	Modified Gamborg B5 medium	1169 ± 89	100.0	ref	*
	BI/MS-macro	BI with macroelements as in MS (R)	531 ± 44	45.4	*	ns
	BI/MS-micro	BI with microelements as in MS (R)	1292 ± 64	110.5	ns	*
	BI/MS-vit	BI with vitamins as in MS (R)	1226 ± 80	104.8	ns	*
	BI/MS-pgr	BI without growth regulators as in R	1279 ± 91	109.4	ns	*
	BI/MS-suc	BI with 2% sucrose as in R	1386 ± 225	118.5	ns	*
	R ^4^	Modified MS medium	404 ± 62	34.5	*	ref
	R/B5-macro	R with macroelements as in B5 (BI)	1322 ± 99	113.0	ns	*
	R/B5-micro	R with microelements as in B5 (BI)	554 ± 44	47.4	*	ns
	R/B5-vit	R with vitamins as in B5 (BI)	485 ± 51	41.5	*	ns
	R/B5-pgr	R with growth regulators as in BI	505 ± 56	43.2	*	ns
	R/B5-suc	R with 3% sucrose as in BI	512 ± 99	43.8	*	ns
Modification of macro- elements	BI	as above	1210 ± 71	100.0	ref	*
	R	as above	656 ± 52	54.3	*	ref
	R+K	R suppl. with 2.35 mM K_2_SO_2_	736 ± 128	60.8	*	ns
	BI/MS-N	BI with nitrogen salts as in MS (R)	753 ± 178	62.2	*	ns
	BI/MS-N+K	BI with nitrogen salts as in MS (R) suppl. with 2.97 mM K_2_SO_2_	736 ± 83	60.8	*	ns
	BI/MS-P	BI with phosphorus salts as in MS (R)	1216 ± 202	100.5	ns	*
	BI/MS-Mg	BI with magnesium salts as in MS (R)	1300 ± 162	107.5	ns	*
	BI/MS-Ca	BI with calcium salts as in MS (R)	1435 ± 107	118.6	ns	*

^1^ %BI—carotenoid content expressed as the percentage of the content in callus growing on the BI medium; ^2^ *P*—significant at *p* < 0.05 (*) or not significant (ns) difference from either BI or R considered as the reference (ref) according to the Dunnett test; ^3^ Modified Gamborg B5 [28] medium; ^4^ Modified MS [29] medium.

**Table 3:** The BI and R media composition with regard to nitrogen content.

N Form	Compound/Ion/N	BI (mM)	R (mM)	BI:R Ratio
Salt	KNO_3_	24.73	18.79	1.3
	(NH_4_)_2_SO_4_	1.01	0	nd ^1^
	NH_4_NO_3_	0	20.61	nd
Ion	NO_3_^−^	24.73	39.41	0.6
	NH_4_^+^	2.02	20.61	0.1
Element	N	26.76	60.02	0.4
Ratio	NO_3_:NH_4_	12.19	1.91	6.4

^1^ not determined

## Data Availability

Not applicable.

## Supplementary Materials

The following are available online at https://www.mdpi.com/article/10.3390/plants10091813/s1, Table S1: Composition of Gamborg B5 and Murashige and Skoog (MS) media and their modified variants, BI and R, respectively; Table S2: Nitrogen salts composition of BI media with a modified N content or NO_3_:NH_4_ ratio; Figure S1: HPLC chromatogram at 452 nm of the sample from callus grown on a BI (control) medium.
