# Supplementary figures and images for: Repression of Carotenoid Accumulation by Nitrogen and NH4+ Supply in Carrot Callus Cells In Vitro
Source: Plants (Basel). 2021 Aug 31;10(9):1813. doi: 10.3390/plants10091813 (PMC8471744; doi:10.3390/plants10091813)

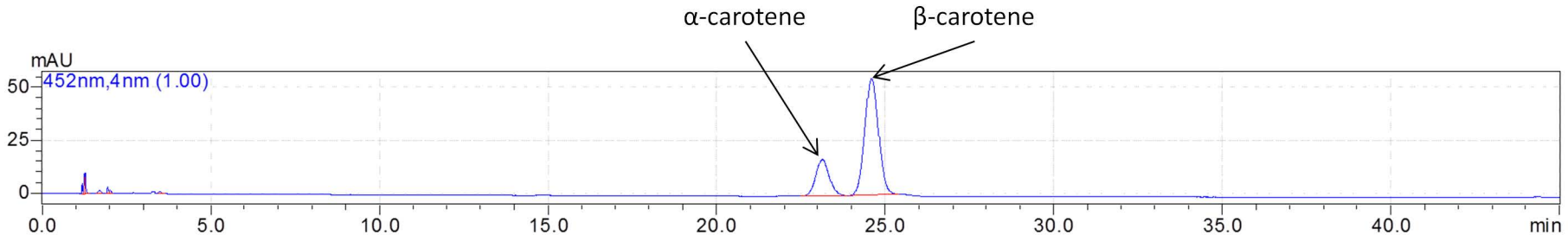

Supplement: Supplementary file 1 [file plants-10-01813-s001.zip › Supplementary Figure S1.pdf]
